# The extraction of natural scene gist in visual crowding

**DOI:** 10.1038/s41598-018-32455-6

**Published:** 2018-09-19

**Authors:** Mingliang Gong, Yuming Xuan, L. James Smart, Lynn A. Olzak

**Affiliations:** 1 Department of Psychology, Miami University, Oxford, OH USA; 2 State Key Laboratory of Brain and Cognitive Science, Institute of Psychology, Chinese Academy of Sciences, Beijing, China; 3 Department of Psychology, University of the Chinese Academy of Sciences, Beijing, China

## Abstract

The gist of natural scenes can be extracted very rapidly and even without focal attention. However, it is unclear whether and to what extent the gist of natural scenes can break through the bottleneck of crowding, a phenomenon in which object recognition will be immensely impaired. In the first two experiments, a target scene, either presented alone or surrounded by four flankers, was categorized at basic (Experiment 1) or global levels (Experiment 2). It was showed that the elimination of high-level semantic information of flankers greatly alleviated the crowding effect, demonstrating that high-level information played an important role in crowding of scene gist. More importantly, participants were able to categorize the scenes in crowding at rather high accuracies, suggesting that the extraction of scene gist might be a prioritized process. To test this hypothesis, in Experiment 3 we compared the crowding effect of three types of stimuli, namely, scenes, facial expressions and letter “E”s. The results showed that scenes could be better categorized than the other two types of stimuli in the crowding condition. This scene gist advantage thus supported our hypothesis. Together, the present studies suggest that scene gist is highly recognizable in crowding, probably due to its prioritization in visual processing.

## Introduction

Objects rarely appear alone in the real world; instead they are usually surrounded by other objects. In peripheral vision, the presence of irrelevant nearby objects greatly deteriorates the recognition of the target object, a phenomenon called visual crowding. To the extent that crowding can be ubiquitous (even when you are reading this sentence), it sets a bottleneck on everyday object recognition in peripheral vision^1–3^, yet it also provides a window into the mechanisms that underlie object perception^2^. For this reason, extensive studies on crowding have been conducted in the last few decades. A commonly reported finding of these studies is that crowding occurs when the target-flanker distance is smaller than 0.5 times the eccentricity of the target^4^, known as “Bouma’s law”.

Crowding has been found in a wide variety of stimuli (for a review, see Manassi and Whitney^5^), ranging from low-level features such as orientation^6,7^, size and hue^8^, to object parts^9,10^ and multiple-feature objects such as letters^4,11–13^, faces^14,15^ and biological motion^16^. For instance, a stronger crowding effect has been shown when an upright face is surrounded by upright faces than inverted ones^15^. This finding suggests that crowding can occur between holistic representations of faces that are represented at higher levels in the visual system. These studies suggest that crowding can occur independently at multiple stages of visual processing^3^ (for a review, see Manassi and Whitney^5^).

Though crowding impairs feature discrimination^6^ and object recognition^14^, it does not impair feature detection^13,17,18^. This finding suggests that crowding may occur in a late stage of visual processing^2^. The recognition of multi-feature objects in crowding can be accommodated by a two-stage model^2,13,17^. Specifically, the detection of target features can be correctly completed in the “feature detection” stage. However, in the following “feature integration” stage, the brain integrates over too large an area around the target due to low spatial resolution^19,20^. This results in features from both the target and flankers pooling together^7,18^. As a consequence, features of the target object fails to be isolated^2^ and the object becomes unrecognizable. According to this theory, high-level (e.g., object-level) information under crowding is lost^3,7,19,21^.

Recent studies, however, have demonstrated that a wide range of high-level information, including word meaning^22,23^, facial expressions^3,24,25^, and digital numbers^26^, survives crowding, even when the information cannot be consciously reported. For example, Kouider *et al*.^24^ showed that participants’ evaluative judgment of a following neutral, unknown Chinese pictograph was biased by the crowded face even when they could not consciously report the emotion of the crowded face. A similar priming effect was also observed from crowded words^23^. More direct evidence comes from Peng *et al*.^22^. They recorded participants’ electrophysiological brain activity while they were performing a semantic relationship judgment task, and it was observed that the N400 component was elicited under crowding, even for participants whose behavioral performance was lower than chance level. Since N400 is an indicator of semantic processing, the finding demonstrates that semantic information did get through the bottleneck of crowding. These studies indicate that high-level information is not entirely lost in crowding; rather, the representation of high-level information can be accomplished despite observers’ inability to consciously access that information^5^.

The meaning or gist of a natural scene includes visual information of all levels (e.g., low-level features, high-level semantic information) and can be defined at conceptual and perceptual levels^27^. Conceptual gist refers to the semantic information that is inferred while viewing a scene. The most commonly inferred concept is at the basic level. For instance, “apple” is a basic-level concept, with “fruit” being its superordinate concept and “Fuji apple” being its subordinate concept. Perceptual gist refers to the image properties (e.g., texture, spatial frequency) that determine the structural representation of a scene. The global properties are a collection of holistic descriptors of scene structure, layout and function^28^. For example, the naturalness or degree of openness of a scene is defined at the global level. Many studies have focused on the conceptual gist (especially gist at the basic level) in the categorization or recognition of natural scenes, yet perceptual gist such as global properties of structure also play an important role^28^. The comparisons between the basic-level gist and the global-level gist have revealed that the categorization of scenes properties at the basic level costs more time than the global level^29–32^, but the basic-level is more preferentially used to describe an object^33,34^. Therefore, one level can show an advantage over the other depending on the specific task.

The gist of a natural scene can be extracted very rapidly^30–32,35^. Li *et al*.^36^ even proposed that this process can be completed in the absence of focal attention. However, this conclusion might not be accurate because participants might be capable of allocating some attention to the scenes^37^. In Li *et al*.’s study, participants were aware that they would be asked about the scene. Given that they were highly trained, it was very likely that participants did distribute their attention to the scene. In fact, direct investigations have shown that the perception of scene gist requires attention^37,38^. The quickly extracted gist can provide a context that guides the allocation of attention toward potential target objects within the scene^1,39–41^. Therefore, gist extraction is vital to the perception of a scene. It should be noted that the extraction of scene gist can be affected by low-level perceptual features such as spatial frequency^42^, color^35,43^, and local phase information^44,45^. Schyns and Oliva^42^ showed that low spatial frequency information seems to dominate early stages of scene gist perception whereas high spatial frequency information is more often used to extract scene gist. However, low-level features are not sufficient for rapid extraction of scene gist; rather, as suggested by the Spatial Envelope Model^46^, the holistic representation (i.e., global configuration) of the scene is vital to its gist perception (see also Greene and Oliva^28^; Oliva and Torralba^47^). Scenes from the same basic-level category tend to share similar spatial structures in terms of openness, expansion and mean depth, etc^46^. Therefore, both low-level features and the holistic representations of scenes inform the gist of a scene.

In the everyday rich and complex surrounding world, different scenes may appear together. For instance, a highway may be located in the middle of and thus crowded by mountains. Will our perception of the highway be impaired when they appear in the periphery of our visual field? Despite extensive studies on the extraction of gist of natural scenes, no study to date, to our knowledge, has ever examined whether and to what extent gist can be extracted under crowding.

To examine the influence of crowding on scene gist extraction, a scene categorization task was employed. In Experiment 1, we explored the extraction of scene gist under crowding at the basic-level (i.e., forests, mountains, highways and tall buildings). In Experiment 2, we examined it at the global-level (i.e., naturalness, indoor/outdoor, openness and expansion). Since both low-level features and the holistic representations of scenes inform scene gist, and crowding can occur independently at multiple stages of visual processing^3^, it is likely that the factors that affect scene gist perception should also affect scene perception under crowding. Specially, flankers may impair the extraction of scene gist either due to the crowding of low-level features or due to the crowding of high-level semantic information or due to both. To disentangle the role of high- and low-level information in the crowding of natural scenes, both intact and scrambled flanker scenes were employed in Experiments 1 and 2. In Experiment 3 we compared the extraction of scene gist with the extraction of facial expression and orientation of letter “E” in the crowding task, aiming to examine which type of information could better break through crowding.

It was predicted that: (1) the presence of surrounding scenes, according to the assumption of crowding, would elicit the crowding effect which impairs the recognition of a scene; (2) both low- and high-level information were playing a role in the crowding of scenes; (3) since high-level information can get through the bottleneck of crowding^3,22–26^, and the gist of a natural scene can be perceived very rapidly^30–32,35^, even in the visual periphery^36,48^, it was reasonable to assume that the gist of a scene could also be perceived even when it was crowded; (4) scene gist could better get through the bottleneck of crowding than facial expression and orientation of letter “E”.

## Experiment 1

This experiment was designed to examine the extraction of scene gist at the basic level. Meanwhile, both intact and scrambled flankers were employed to examine the crowding between high-level information of scenes as well as the crowding between low-level features. Scrambled scenes are control stimuli whose semantic information are removed while their low-level visual features are preserved. If both low- and high-level of information play their roles in crowding, greater crowding effects should be observed when the target scene is crowded by intact flankers in comparison to by scrambled flankers.

### Methods

#### Participants

Sixty-two undergraduate students (24 males, mean ages = 19.2) participated in this experiment for research credit. Participants were randomly assigned to one of three conditions. Twenty-three participants were assigned to the intact flanker condition, twenty-two were assigned to the phase-scrambled flanker condition, and seventeen were assigned to the diffeomorphed flanker condition. All participants had normal or corrected-to-normal vision. The present and following experiments were approved by the Institutional Review Board (IRB) of Miami University, and were carried out in accordance with the relevant guidelines and regulations. Informed consent was obtained from all participants before running the study.

#### Stimuli

A total of 800 full-color images were selected from a large laboratory database (http://cvcl.mit.edu/database.htm)^46^. The images belonged to four basic-level categories, including natural (forests and mountains) and man-made scenes (highways and tall buildings). Each category contained 200 images. The images were viewed at a distance of around 57 cm, with a visual angle of 4° × 4° for each image (target and flanker). Target eccentricity was randomly chosen from three visual angles (9°, 11°, or 13°). The target image was either presented alone or surrounded by four flankers. The visual angle of target-flanker separation (center to center) was 4.5°, which was not larger than half of either eccentricity, thus crowding was supposed to occur at each eccentricity according to Bouma’s law.

**Figure 1:**
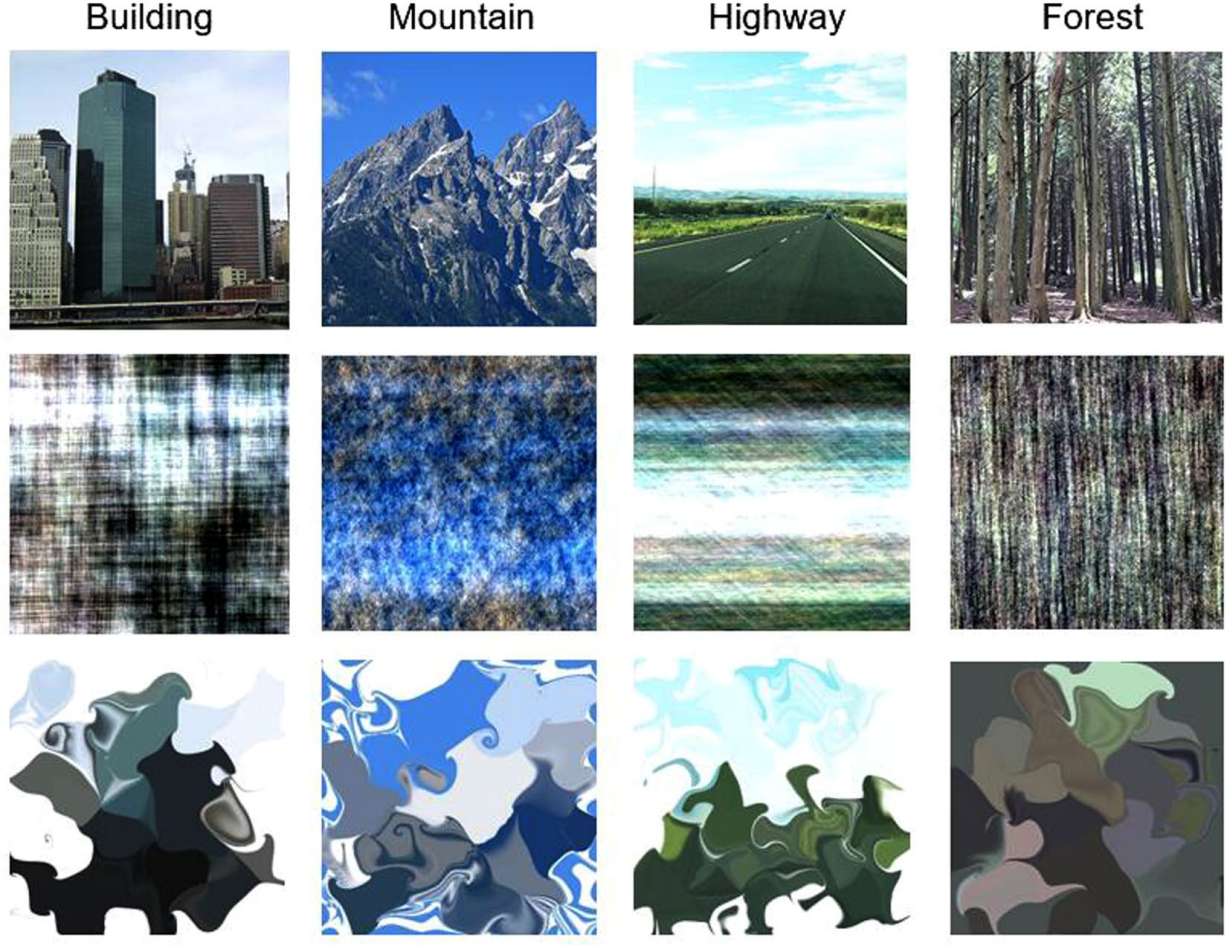
Examples of scenes used in Experiment 1. The scenes belong to four basic-level categories (i.e., forest, mountain, highway and tall building). The upper panel shows intact images, the middle and lower panels show corresponding phase-scrambled and diffeomorphed versions, respectively. The images in the first row were adapted from photos taken by the first author and were not the actual images used in the study.

The experiment consisted of three conditions that differed in the type of flankers. In the intact-flanker condition, flankers were four normal/intact images presented at four locations (left, right, above and below of the target). The two scrambled-flanker conditions were identical to the intact-flanker condition, except that all intact flankers were scrambled via phase-scrambling and diffeomorphic transformation^49^. The phase-scrambling method scrambles the intact images in the Fourier phase domain and has been extensively used to create control (meaningless) stimuli^47^. However, phase-scrambling might distort the basic visual properties of scene images^49^. For instance, phase coherence contained in intact images could be altered due to phase-scrambling^50^. Since phase coherence is vital to perceptual information such as edges and contours^51^ that are responsible for the similarity between targets and flankers, phase-scrambling may alter crowding. This alteration derives from the change of low-level features rather than the change of gist. Hence, they proposed another method, diffeomorphic transformation, to create control stimuli. The diffeomorphic transformation generates a set of two-dimensional cosine components with random phase and amplitude which repeatedly apply a flow field, forming a one-to-one mapping between the original and transformed spaces without duplicating or removing any parts. Therefore, diffeomorphic transformation can better preserve the basic perceptual properties of the image while removing meaning (for more details, see Stojanoski & Cusack^49^). Figure 1 shows the example images used in the three conditions.

#### Design and Procedures

A 3 (Flanker type: intact vs. phase-scrambled vs. diffeomorphed) × 3 (Eccentricity: 9° vs. 11° vs. 13°) mixed design was employed, with flanker type being a between-subject factor and eccentricity being a within-subject factor. Each participant was assigned to one of the three flanker type conditions and completed the task for all three eccentricities in both crowded and uncrowded conditions. Before the formal experiment, each participant was familiarized with the four categories (i.e., forest and mountain, highway and tall building) by viewing sample images of each category. Then they completed four blocks, one for each category (e.g., mountain). At the beginning of each block, a basic-level category (e.g., mountain) was specified and participants were told to decide whether the images presented within the block belonged to the category or not by pressing either “F” or “J” on the keyboard as accurately and quickly as possible in 3000 ms. Half of the participants were instructed to press “J” if the target belonged to the specified category and press “F” if the target did not belong to the category. The assigned keys for the other half participants were reversed. Each block consisted of 12 practice trials and 160 experimental trials and the order of the four blocks was randomized.

**Figure 2:**
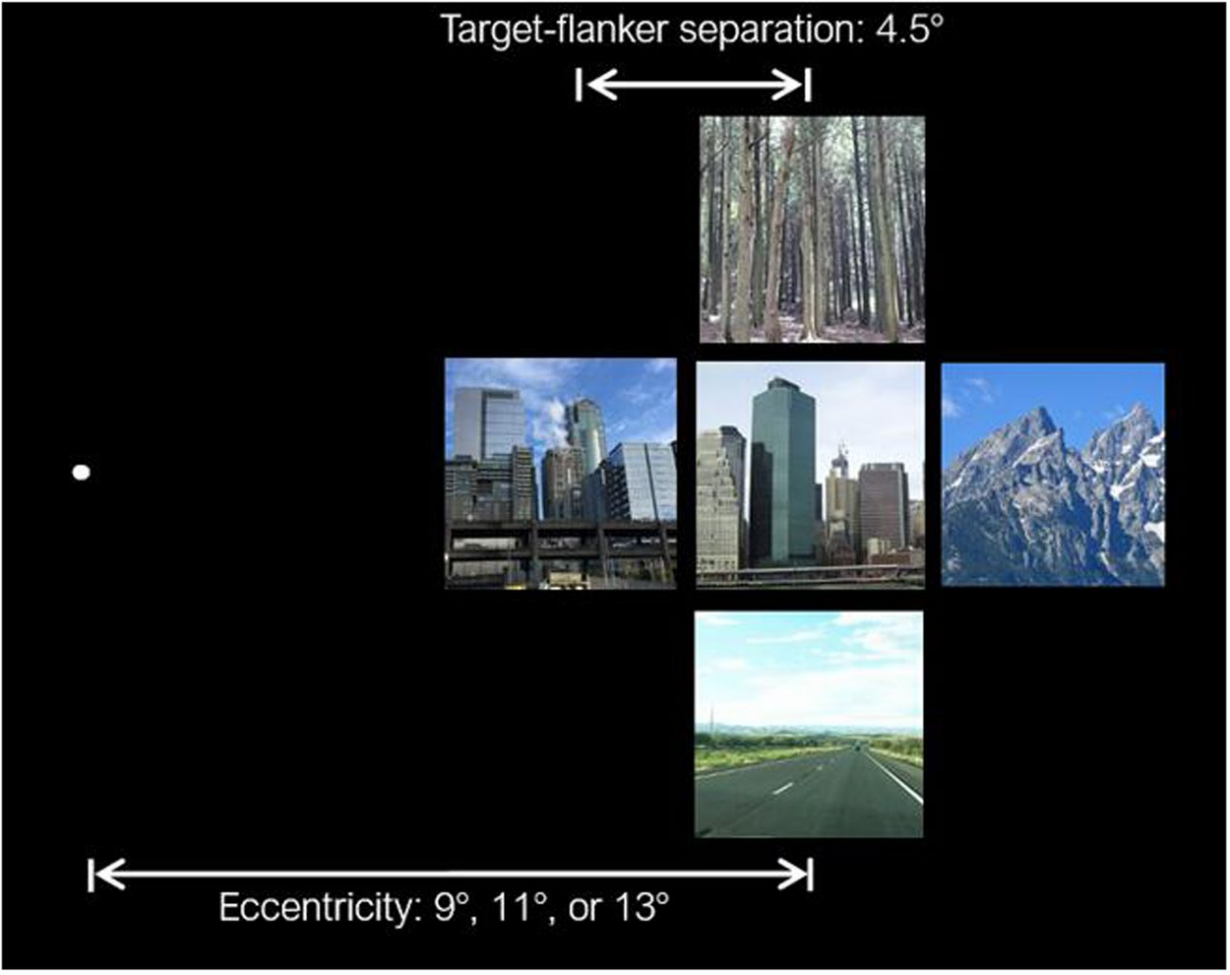
Sample array of the stimuli. The central image is the target, surrounded by four flanker scenes. In the experiment, the fixation point was presented at the center of the screen. The images were adapted from photos taken by the first author and were not the actual images used in the study.

On each trial, a fixation point was displayed at the center of the screen for 500 ms, followed by the stimulus, either a single target or a target with four flankers, appearing randomly on the left or right side of the fixation for 100 ms (see Fig. 2). The brief presentation time and random location of the target could avoid eye movement toward the target since a minimum of 150–200 ms is required to saccade to an unexpected stimulus^52^. During this period, the fixation point remained on the screen; participants were asked to keep their gaze on it. After that, the stimuli and the fixation were replaced by a blank screen, and the participants were instructed to complete the categorization task. The entire session lasted about 35 minutes. To minimize the effect of fatigue, the participants were encouraged to take a break in the interval of the blocks.

### Results

**Figure 3:**
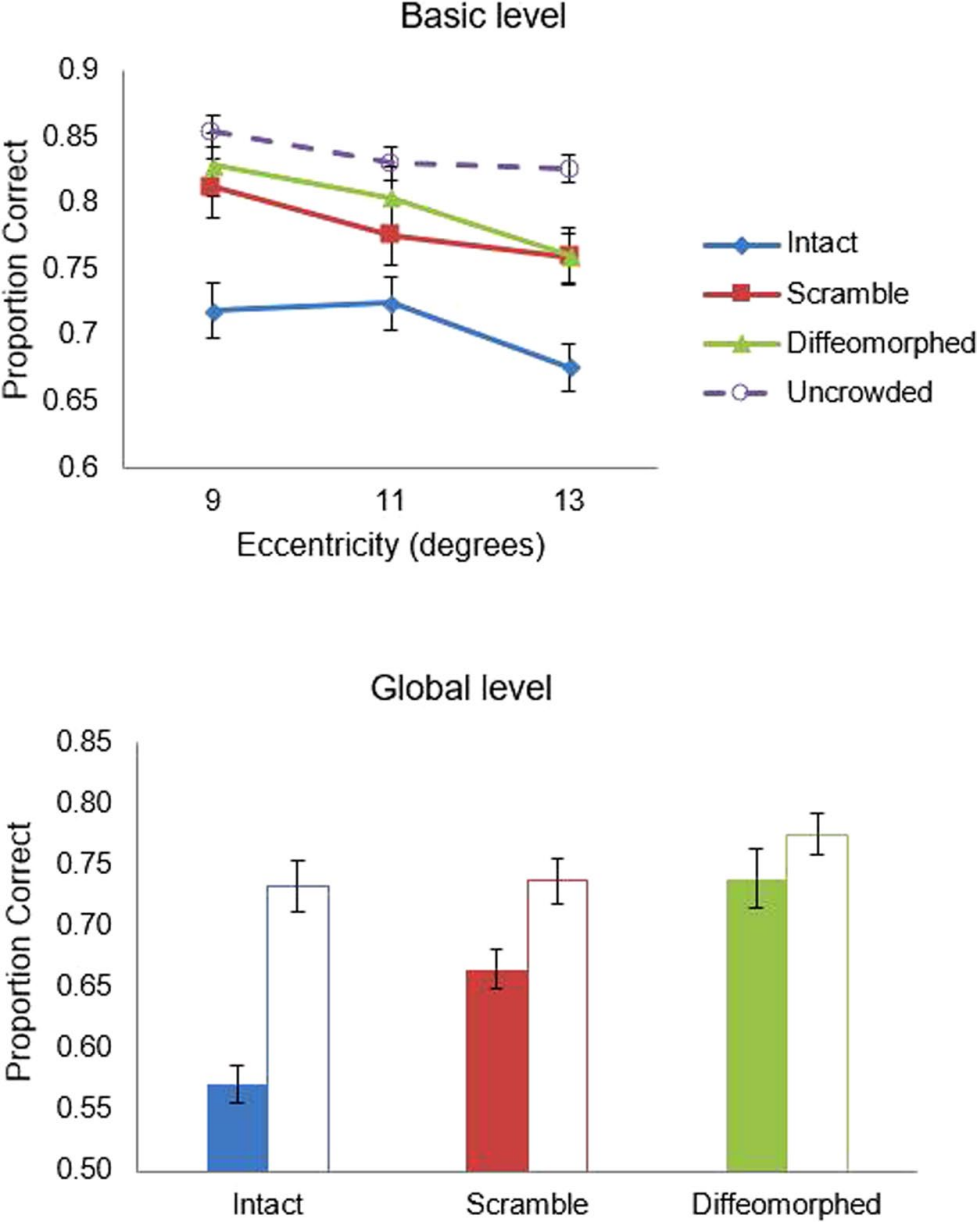
Results of Experiments 1 and 2. The graph in the top panel shows the proportion of correct categorization of basic-level gist as a function of flanker type and eccentricity in Experiment 1, wherein the dashed line indicates the proportion of correct categorization for the uncrowded condition averaged across all trials. The graph in the bottom panel shows the proportion of correct categorization of global-level gist in Experiment 2. The solid bars indicate the proportion of correct categorization for the crowded condition and the hollow bars indicate the proportion of correct categorization for uncrowded condition. Error bars indicate ±1 SEM.

Percentage of correct trials was computed both when there were no flankers (uncrowded condition) and when there were flankers (crowded condition). Results are plotted in the top panel of Fig. 3.

First, a 2 (Crowding: crowded vs. uncrowded) × 3 (Eccentricity: 9° vs. 11° vs. 13°) ANOVAs were conducted for each flanker type condition. We did the ANOVAs separately since flanker type was a between-subjects factor. When flankers were intact, the main effect of crowding was significant, *F* (1, 22) = 81.24, *p* < 0.001, ${\eta }_{{\rm{p}}}^{2}$ = 0.79. Consistent with our prediction 1, participants categorized the target image with a significantly higher accuracy when it was uncrowded (*M* = 0.82) than when it was crowded (*M* = 0.71). The main effect of eccentricity was significant, *F* (2, 44) = 6.00, *p* = 0.005, ${\eta }_{{\rm{p}}}^{2}$ = 0.21. Comparisons with Bonferroni adjustment showed that accuracy was not significantly different between the eccentricity of 9° and 11°, but both eccentricities were significantly higher than the eccentricity of 13°. The interaction between eccentricity and crowding was not significant, *F* (2, 44) = 2.45, *p* = 0.098, ${\eta }_{{\rm{p}}}^{2}$ = 0.10.

When flankers were phase-scrambled, the main effect of crowding was significant, *F* (1, 21) = 51.46, *p* < 0.001, ${\eta }_{{\rm{p}}}^{2}$ = 0.71, with the proportion correct being significantly higher when the target was uncrowded (*M* = 0.85) than when it was crowded (*M* = 0.78). The main effect of eccentricity was significant, *F* (2, 42) = 9.60, *p* < 0.001, ${\eta }_{{\rm{p}}}^{2}$ = 0.31. Comparisons with Bonferroni adjustment showed that accuracy at the eccentricity of 9° was significantly higher than the accuracy at the eccentricity of 11° or 13°, and no significant differences was shown between the eccentricity of 11° and 13°. The interaction between eccentricity and crowding was not significant, *F* (2, 42) = 1.25, *p* = 0.297, ${\eta }_{{\rm{p}}}^{2}$ = 0.06.

When flankers were diffeomorphed, the main effect of crowding was significant, *F* (1, 16) = 38.54, *p* < 0.001, ${\eta }_{{\rm{p}}}^{2}$ = 0.71, with the proportion correct being significantly higher when the target was uncrowded (*M* = 0.85) than when it was crowded (*M* = 0.80). The main effect of eccentricity was significant, *F* (2, 42) = 10.18, *p* < 0.001, ${\eta }_{{\rm{p}}}^{2}$ = 0.39. Comparisons with Bonferroni adjustment showed that accuracy at the eccentricity of 9° was significantly higher than the accuracy at the eccentricity of 11° or 13°, and no significant differences was shown between the eccentricity of 11° and 13°. The interaction between eccentricity and crowding was not significant, *F* (2, 32) = 2.11, *p* = 0.138, ${\eta }_{{\rm{p}}}^{2}$ = 0.12.

Furthermore, the accuracies at all three eccentricities were higher than chance level (0.5) for intact flankers (9°: 71.9%, 11°: 72.4%, 13°: 67.6%), phase-scrambled flankers (9°: 81.2%, 11°: 77.6%, 13°: 75.9%) and diffeomorphed flankers (9°: 82.9%, 11°: 80.4%, 13°: 76.0%). These results are consistent with our prediction 3, showing that participants can perceive the target scenes quite well even when they were crowded.

Then a 3 (Flanking conditions: intact vs. phase-scrambled vs. diffeomorphic transformation) × 3 (Eccentricity: 9° vs. 11° vs. 13°) analysis of variance (ANOVA) was conducted on accuracy. The main effect of flanker type was significant, *F* (2, 59) = 4.75, *p* = 0.012, ${\eta }_{{\rm{p}}}^{2}$ = 0.14. Tukey’s HSD test revealed that accuracy in the intact flanker condition was significantly lower than accuracy in the phase-scrambling condition and diffeomorphed flanker condition, *p* = 0.044 and *p* = 0.020, and no significant difference between the latter two conditions, *p* = 0.885. The main effect of eccentricity was also significant, *F* (2, 118) = 38.41, *p* < 0.001, ${\eta }_{{\rm{p}}}^{2}$ = 0.40. Comparisons with Bonferroni adjustment showed that accuracy significantly decreased with the increase of eccentricity (*p*s < 0.001). The interaction between flanker type and eccentricity was not significant, *F* (4, 118) = 2.42, *p* = 0.052, ${\eta }_{{\rm{p}}}^{2}$ = 0.08.

### Discussion

The first experiment showed that the presence of flankers significantly reduced accuracy, which suggested that there was a crowding effect that significantly impaired basic-level scene categorization. However, the accuracies at all three eccentricities were significantly higher than chance level (0.5) even though the stimuli were only presented for 100 ms. This indicates that the gist of a scene at the basic level, a type of semantic information, was still greatly extracted even when the scene was crowded by other scenes.

The experiment also showed a significant crowding effect using scrambled (phase-scrambled and diffeomorphed) flankers, a type of stimuli containing only low-level physical properties. This result suggests that low-level physical properties can crowd the categorization of scenes, in line with other studies^6,8^. More importantly, the experiment also showed a better performance (i.e., higher accuracy) when a target was crowded by scrambled flankers than crowded by intact flankers. That is, the crowding became significantly weaker when the semantic information was removed via scrambling. This finding seemed to indicate that high-level semantic information of surrounding flankers was also playing a role in producing the crowding effect, i.e., there was a crowding effect between the high-level semantic information of scenes. However, it is worth noting that the two scrambling methods especially the phase scrambling may also alter low-level structures, we thus should be cautious when drawing a solid conclusion about the role of high-level semantic information. Considering that crowding has been shown to occur between the high-level information of target and flankers for stimuli such as faces^15,25^ and biological motion^16^, it is very likely that crowding can occur between the high-level information of scenes.

## Experiment 2

Experiment 1 showed that crowding significantly impaired scene categorization at the basic level, yet the gist of a scene could still be largely extracted. In addition to the basic level, the gist of a scene can also be defined at a global level^27^. It has been found that human observers perceive the two levels of properties differently: Basic-level properties are named more quickly^53^, but categorized slower than global-level properties^30^. In these studies, objects were presented in the fovea, and it would be intriguing to know whether the extraction of scene gist at the global level is advantageous, disadvantageous, or similar to the extraction at the basic level in a visual crowding task. More importantly, we would like to know whether the pattern of results (e.g., extraction of gist in crowding, crowding between low- and high-level information) observed in basic-level gist in Experiment 1 would be replicated in global-level gist. Experiment 2 was designed to investigate these questions.

### Methods

#### Participants

Sixty-one undergraduate students (34 males, mean age = 19.7) participated in this experiment for research credit. Twenty-two participants were randomly assigned to intact flanker condition, twenty-two were assigned to phase-scrambled flanker condition and seventeen were assigned to diffeomorphed flanker condition. All participants had normal or corrected-to-normal vision and provided informed consent before running the experiment.

#### Stimuli

**Figure 4:**
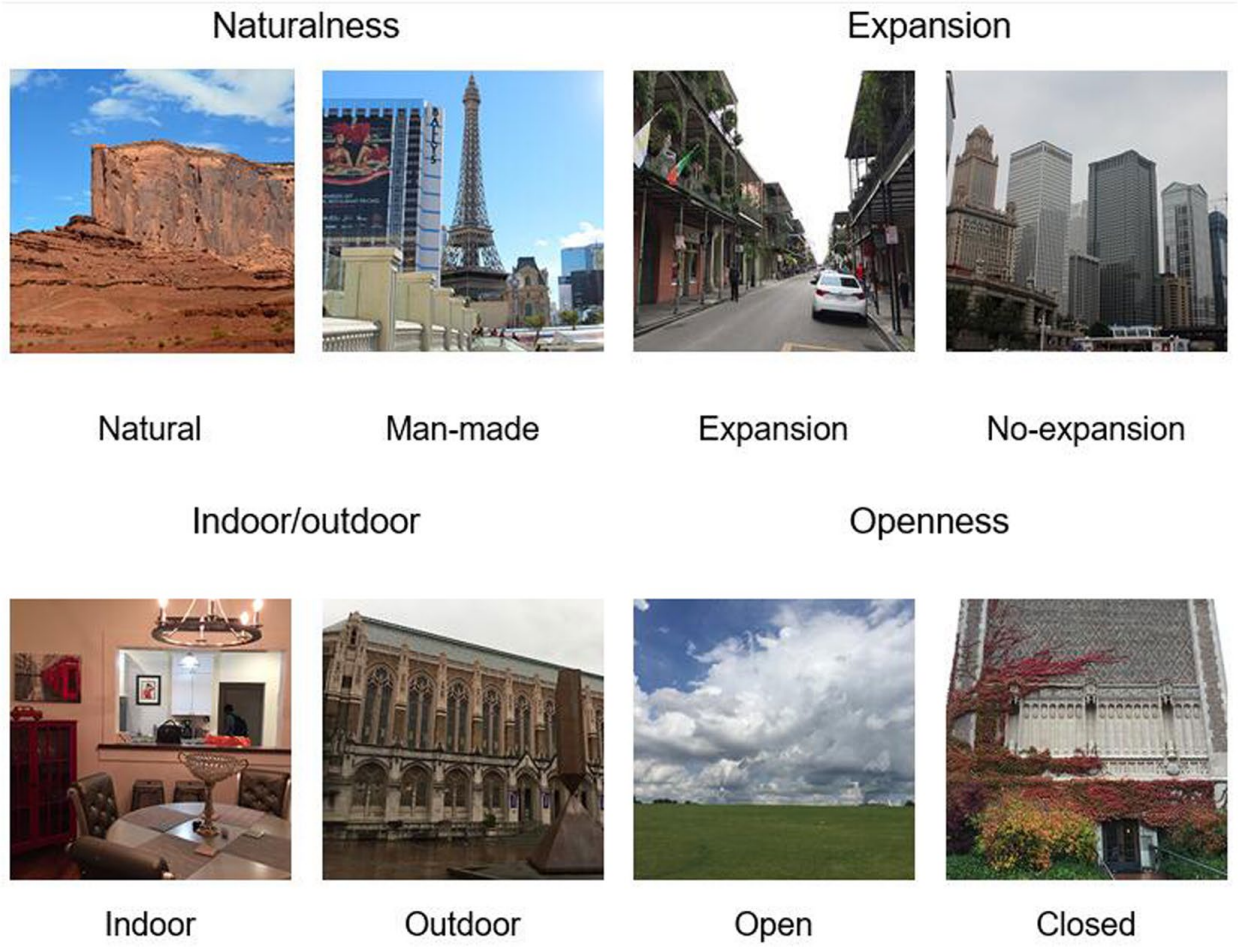
Examples of scenes used in Experiment 2. The scenes belong to four global properties (naturalness, openness, expansion and indoor versus outdoor). Each property has two poles (e.g., natural vs. man-made for naturalness). The images were adapted from photos taken by the first author and were not the actual images used in the study.

A total of 800 full-color images were selected from the same laboratory database that had been previously rated by human observers along each of the global properties^30^. The images exhibited four global properties (naturalness, expansion, indoor/outdoor and openness), with each property having two poles (natural vs. man-made, expansion vs. non-expansion, indoor vs. outdoor and open vs. close, respectively). Each property contained 200 images (100 for each pole). See Fig. 4 for example images. Image size and target-flanker separation were the same as those in Experiment 1. Likewise, two types of scrambled versions (i.e., phase-scrambled and diffeomorphed) of the flankers was employed to examine the role of low- versus high-level information in the crowding of global scene properties.

#### Design and Procedures

Most of the procedures were the same as Experiment 1, yet there were several differences. First, the eccentricity of target was fixed at 11° and the number of trials in each block was reduced to 80. Second, at the beginning of each block, a global property (e.g., naturalness) was specified and participants were instructed to decide whether the target belonged to one pole or the other. Thus the task was actually changed from a “Yes or No” question (e.g., “is the target a mountain?”) in Experiment 1 to an “A or B” question (e.g., “is the target a natural or man-made scene?”), though in essence they were the same. The entire session lasted about 20 minutes.

### Results

#### Global-level gist

Again, percentage of correct recognition of the gist at the global level was computed. The results are shown in the bottom panel of Fig. 3. First, paired t-tests were conducted to compare the accuracy between the crowded condition and uncrowded condition for each flanker type. Results revealed that scene gist was always significantly better recognized in the uncrowded condition relative to the crowded condition [intact flankers: *t* (21) = 9.06, *p* < 0.001; phase-scrambled flankers: *t* (21) = 5.27, *p* < 0.001, diffeomorphed flankers: *t* (16) = 2.35, *p* = 0.032]. Analyses also showed that the accuracies in all conditions were significantly higher than the chance level (all *p*s < 0.001).

Then a one-way ANOVA was conducted to examine the effect of flanker type on the categorization of scene gist in the crowded condition, which yielded a significant main effect of the flanker type, *F* (2, 58) = 21.72, *p* < 0.001, ${\eta }_{{\rm{p}}}^{2}$ = 0.43. Tukey’s HSD test showed that the accuracy was significantly higher when flankers were phase-scrambled and diffeomorphed than when they were intact (*ps* < 0.002), and the accuracy was significantly higher for diffeomorphed flankers than phase-scrambled ones (*p* = 0.016).

#### Basic-level vs. global-level gist

**Figure 5:**
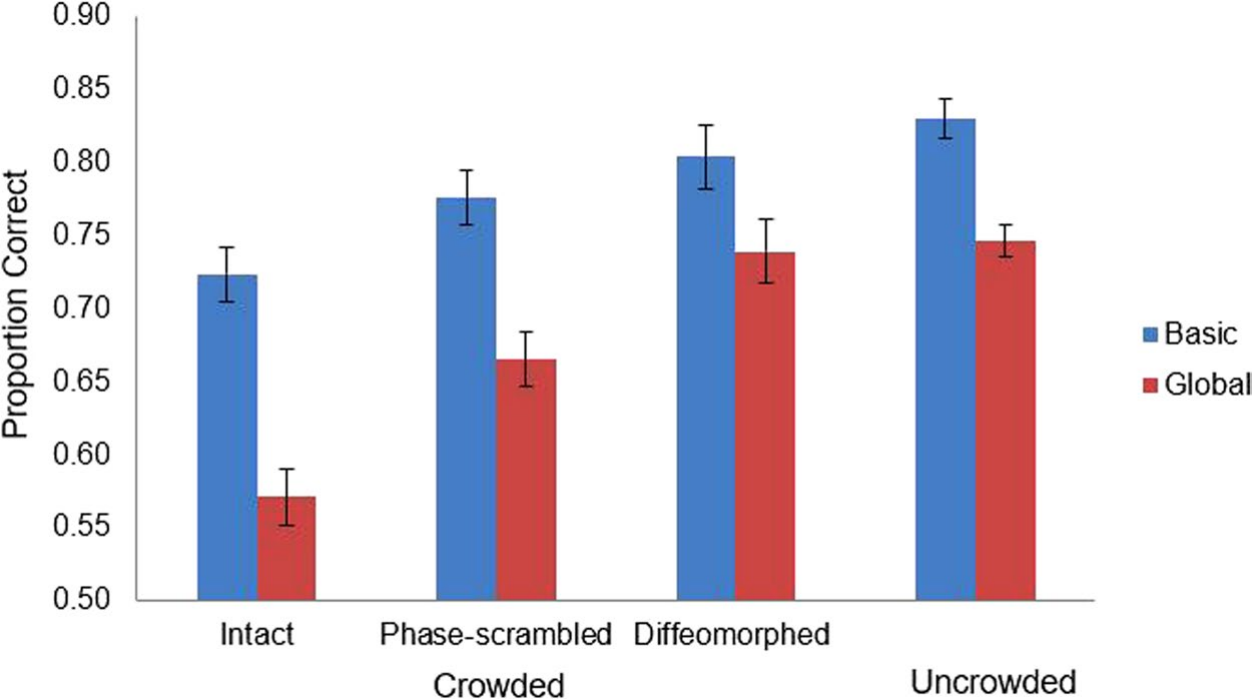
The graph shows the proportion of correct categorization of basic-level gist versus global-level gist in different conditions. Error bars indicate ±1 SEM.

Results are plotted in Fig. 5. Stimuli in Experiment 2 were presented at an eccentricity of 11°. To compare the performance on basic level and global level, we only used the basic-level data at the eccentricity of 11°. For the uncrowded condition, a paired t-test was conducted to compare the proportion correct between the basic level and global level, which yielded a significant result, *t* (122) = 10.78, *p* < 0.001.

A 2 (Gist level: basic vs. global) × 3 (Flanker type: intact vs. phase-scrambled vs. diffeomorphic transformation) ANOVA was conducted on proportion correct in the crowded condition, which yielded a non-significant interaction between gist level and flanker type, *F* (2, 117) = 2.40, *p* = 0.095, ${\eta }_{{\rm{p}}}^{2}$ = 0.04. The main effect of gist level was significant, *F* (1, 117) = 45.70, *p* < 0.001, ${\eta }_{{\rm{p}}}^{2}$ = 0.28, with basic level (*M* = 0.76, *SD* = 0.10) being more accurately categorized than global level (*M* = 0.65, *SD* = 0.10). The main effect of flanker type was also significant, *F* (2, 117) = 19.59, *p* < 0.001, ${\eta }_{{\rm{p}}}^{2}$ = 0.25. Tukey’s HSD test showed that accuracy was significantly higher when flankers were phase-scrambled (*M* = 0.72, *SD* = 0.09) and diffeomorphed (*M* = 0.65, *SD* = 0.10) than when they were intact (*M* = 0.77, *SD* = 0.09), *p* = 0.001 and *p* < 0.001, respectively, and the accuracy was higher for diffeomorphed flankers relative to phase-scrambled flankers, *p* = 0.035.

### Discussion

Experiment 2 replicated most of the findings in Experiment 1. First, the presence of flankers significantly reduced the accuracy on the categorization of global-level properties, suggesting that there was a crowding effect. Second, the accuracy was very high (significantly higher than chance level) for both intact and scrambled flankers, indicating that the gist of a scene at the global level could still be extracted to a significant extent in crowding. Third, the experiment also showed a stronger crowding effect for intact flankers than scrambled ones, demonstrating that high-level information was also playing a crucial role in crowding.

The comparison of the results of Experiment 1 and Experiment 2 showed that the extraction of scene gist at the global level is harder than that at the basic level. This is in accordance with the research that showed an advantage for the categorization of basic-level properties relative to the categorization of global properties when a single scene image was presented in far periphery^48^. It is worth noting that the accuracy for the recognition of scene gist, either at the basic level or at the global level, was very high. Given that the stimuli were presented for only 100 ms and that the target scene was surrounded by four flankers in the experiments, this finding is very striking. We postulate that the extraction of scene gist is a prioritized and largely automatic process. To test this postulation, it is necessary to compare the extraction of scene gist with the extraction of other information to examine whether there is an advantage for the extraction of scene gist.

## Experiment 3

This study was designed to examine whether the extraction of scene gist is a prioritized process. To this end, we compared the extraction of scene gist to the extraction of other information including facial expression and orientation of letter “E”. Facial expression was chosen because it has been shown that faces are specially processed (i.e., there are brain areas that are specialized for face perception)^54^, and facial expression is a commonly studied form of high-level information in crowding. The orientation of the letter “E” was chosen because it is widely used in eye examinations, and orientation discrimination is a commonly used lower-level vision task in crowding. Here we employed similar crowding tasks to examine how crowding effect varied with stimulus type and eccentricity.

### Method

#### Participants

Sixty-three undergraduate students (21 males, mean ages = 18.76) participated in this experiment for research credit, with twenty-one in each eccentricity condition. All had normal or corrected-to-normal vision. All participants provided informed written consent before running the experiment.

Stimuli: Three different types of stimuli were employed:

Scenes: Eighty scene images were selected from the same database^46^ as Experiment 1. They belonged to the four basic categories (building, forest, highway and mountain), with each category consisting of 20 images of different identities.

Faces: Eighty White male real faces were selected from the Chicago Face Database^55^. This database consisted of high-resolution, standardized photographs. Each photograph was cropped so that it included only the head and was standardized in size (4 degrees in width and 4 degrees in height). The faces belonged to four facial expression categories (happy, angry, fear and neutral), with each expression category consisting of 20 images of different identities.

Letters: Letter “E” was frequently used for vision examination and was employed in this experiment. The orientation of the letters consisted of four facing directions (up, down, left, right), with an upright “E” being facing right. Like scenes and facial expressions, each category of the facing directions consisted of 20 different font types.

Each type of stimuli consisted of four categories (i.e., four scene categories, four facial expressions and four facing directions) which corresponds to four potential choices. Stimuli of different types were presented in blocks and the order of the blocks was randomized. Within each block, the target image was either presented alone or surrounded by two flankers. Image size (4° × 4° in visual angel) and target-flanker separation (4.5° from center to center) were the identical across stimuli type and were the same as Experiments 1 and 2.

#### Design and Procedures

Visual crowding tasks were used to compare the extraction of scene gist with the extraction of facial expression and the extraction of orientation of letter “E”. The design was a 2 (crowding: crowded vs. uncrowded) × 3 (stimulus type: scene vs. face vs. letter) × 3 (eccentricity: 9° vs. 13° vs. 20°) mixed design, with the eccentricity being a between-subject factor and the other two being within-subject factors. Each participant completed all three tasks (i.e., scene categorization task, facial expression categorization task, letter direction discrimination task) at the eccentricity of either 9°, 13°, or 20°. In the scene categorization task, the participants were asked to categorize scene pictures into building, forest, highway or mountain. In the facial expression categorization task, the participants were demanded to categorize faces into angry, fear, happy or neutral. In the orientation discrimination task, the participants were required to determine whether the facing direction of letter “E” was left, right, up or down. Each task was in its own block and the order of the blocks was randomized. Each block consisted of 12 practice trials and 96 experimental trials. In total, each participant needed to complete 288 experimental trials.

On each trial, a fixation cross was displayed in the center of the screen for 1000 ms, followed by the stimuli that appeared at one of the eccentricities either on the left or right side of the fixation for 100 ms. In the uncrowded condition, the stimulus was an isolated target without any flanker; in the crowded condition, the stimuli consisted of a target and two flankers. During the stimuli presentation period, the fixation cross remained on the screen, with participants’ gaze staying on it. After that, the stimuli and the fixation were replaced by a blank screen, and the participants were instructed to finish the corresponding tasks by pressing the appropriate keys on a standard keyboard as accurately as possible. To minimize the effect of fatigue, the participants were encouraged to take a break in the interval of blocks.

### Results

**Figure 6:**
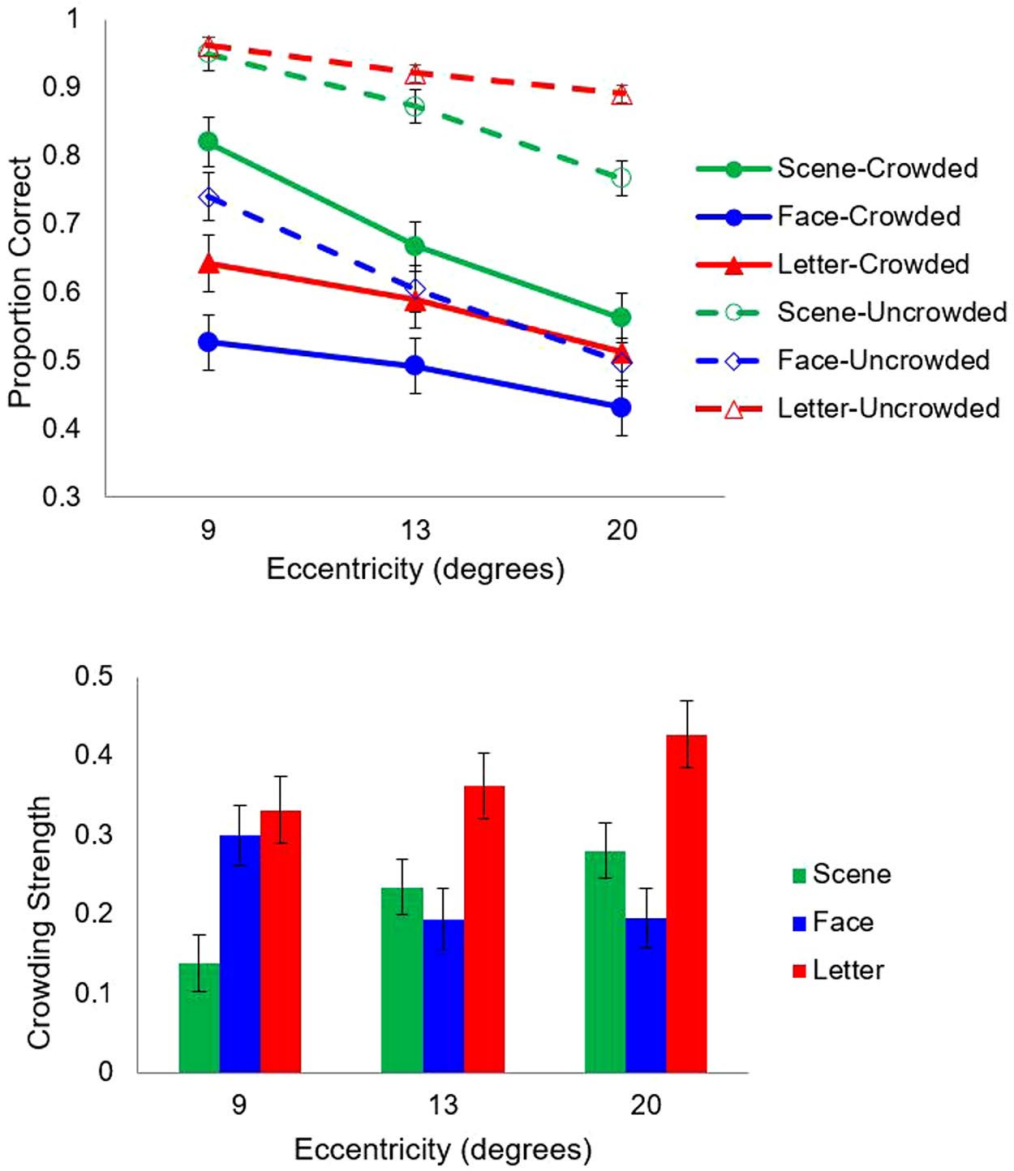
Results of Experiment 3. The two graphs show proportion correct (above) and crowding strength (below) as a function of eccentricity and flanker type. Error bars indicate ±1 SEM.

Percentage of correct trials was computed both when there were no flankers (uncrowded condition) and when there were flankers (crowded condition). Results are plotted in Fig. 6.

A three-way repeated measures ANOVA was conducted on accuracy. Results showed a significant main effect of crowding, *F* (1, 60) = 217.66, *p* < 0.001, ${\eta }_{{\rm{p}}}^{2}$ = 0.78, with crowded condition (*M* = 0.59, *SD* = 0.17) yielding a significant lower accuracy than uncrowded condition (*M* = 0.82, *SD* = 0.11). The main effects of stimulus type and eccentricity were also significant, *F* (1, 60) = 6.73, *p* = 0.002, ${\eta }_{{\rm{p}}}^{2}$ = 0.75 and *F* (1, 60) = 177.49, *p* < 0.001, ${\eta }_{{\rm{p}}}^{2}$ = 0.18, respectively. Pairwise comparisons showed that the accuracy at the eccentricity of 20° was significantly lower than the accuracy at the eccentricity of 9° (*p* = 0.002), and no significant difference was found between other eccentricities. The three-way interaction was also significant, *F* (4, 120) = 7.47, *p* < 0.001, ${\eta }_{{\rm{p}}}^{2}$ = 0.20, so we did follow-up analyses. Results showed that the accuracy in the scene gist categorization task was always significantly higher than the accuracies in the other two tasks at all eccentricities in the crowded condition, *p*s < 0.05. However, the accuracy in the scene gist categorization task was not always significantly higher (actually sometimes lower) than the accuracies in the other two tasks in the uncrowded condition. Specifically, the accuracy in the scene gist categorization task was significantly lower than the accuracy in the orientation discrimination task at the eccentricities of 13° and 20°, *p*s < 0.001, but the two did not differ at the eccentricity of 9°. The accuracy in the scene gist categorization task and orientation discrimination task was always higher than the accuracy in the facial expression categorization task, *p*s < 0.001.

The results suggested that scene gist, facial expression and letter orientation all suffered from crowding but to different degrees. However, it was not really known how “crowded” each type of stimuli was since the difficulty of each task in the uncrowded condition was not matched. To illustrate which type of stimulus suffered the most from crowding and which could best break through crowding, we calculated the crowding strength using Equation (1):

1 $${\rm{Crowding}}\,{\rm{strength}}=\frac{Uncrowded-Crowded}{Uncrowded}$$

where the numerator is the difference between the accuracy in the uncrowded condition and the accuracy in crowded condition, and the denominator is the accuracy in the uncrowded condition that serves as the baseline.

A 3 (stimulus type: scene vs. face vs. letter) × 3 (eccentricity: 9° vs. 13° vs. 20°) ANOVA was conducted on crowding strength. Results showed a significant main effect of stimulus type (*F* (2, 120) = 33.74, *p* < 0.001, ${\eta }_{{\rm{p}}}^{2}$ = 0.36) and insignificant main effect of eccentricity (*F* (2, 60) = 0.55, *p* = 0.578, ${\eta }_{{\rm{p}}}^{2}$ = 0.02). The interaction between stimulus type and eccentricity was significant, *F* (2, 120) = 7.15, *p* < 0.001, ${\eta }_{{\rm{p}}}^{2}$ = 0.19. Post-hoc analyses revealed that the crowding strength of scene gist was always significantly weaker than the crowding strength of letter orientation at all three eccentricities (*p*s < 0.001), suggesting that scene gist could better get through the bottleneck of crowding. Compared with the crowding strength of facial expression, the crowding strength of scene gist was significantly weaker at the eccentricity of 9° (*p* < 0.001), similar at the eccentricity of 13° (*p* = 0.238), and significantly stronger at the eccentricity of 20° (*p* < 0.001).

### Discussion

In this study we compared the extraction of scene gist with the extraction of facial expression and orientation of letter “E”. We found that all three types of stimuli were largely recognized, especially when the eccentricity was small. Given that crowding greatly impairs information fidelity, this result indicates that people can recognize image contents with only very little information. In reality, it has been shown that image contents can be reliably categorized when the images (of scenes, faces, and birds) are reduced down to a single pixel^56^. On the one hand, scene gist was always better extracted than facial expression and letter orientation when they were crowded, though it was not always the case when they were uncrowded. This finding suggested that scene gist could better get through the bottleneck of crowding. On the other hand, the crowding strength of scene gist was always weaker than letter orientation, and it was also weaker than facial expression at small eccentricity (i.e., 9°) but stronger at large eccentricity (i.e., 20°). One explanation could be that there was a floor effect for the recognition of facial expression at the eccentricity of 20°, so the presence of flankers did not harm the extraction of facial expression as much as they harmed the extraction of scene gist. Together, the study showed that scene gist could better get through the bottleneck of visual crowding compared to facial expression and letter orientation, which supported our hypothesis that the extraction of scene gist was a prioritized process.

## General Discussion

The first two experiments showed a crowding effect between gist of adjacent scenes at both basic and global levels. Furthermore, we showed that both low-level physical properties and high-level semantic information were playing roles in crowding, suggesting that crowding of scene gist occurs at multiple stages in the visual system. More importantly, we showed that the gist of a scene, albeit crowded, could still be greatly extracted at both basic and global levels.

Despite the many studies that have been done on crowding, the exact mechanisms underlying crowding are still unknown. For instance, it is unclear where crowding takes place. Substantial work has been done to investigate this “where” question, and mixed results have been shown. Many studies have shown that crowding happens in early visual cortex (See Levi^2^). For example, Yu *et al*.^57^ showed that the crowding effects for different word configurations (upright, scrambled, horizontal-flip, vertical-flip and letter flip) were similar, suggesting that crowding occurred at the feature level. However, a growing body of evidence indicates that crowding can also occur at higher levels in the visual system^9,14,15,58^. For instance, the “holistic crowding” between faces demonstrates that crowding can occur between configural representations (i.e., representations of relations between features) of faces^9,15^. In the present study, we examined the locus of crowding with stimuli of scene images. To create flankers with only low-level physical features, we scramble the flankers via phase-scrambling and diffeomorphic transformation to eliminate semantic information. The results showed a stronger crowding effect with intact flankers compared to scrambled flankers, suggesting that, in addition to low-level crowding, there was also a scene-based high-level crowding. This finding provided further support to the claim that crowding occurs at multiple levels in the visual system^5,9,14,15,59^ with stimuli of scenes images.

It is well established that the gist of a natural scene can be extracted very rapidly^30–32,35^. This process can even be completed in the visual periphery^36,60^, extending as far as a 70° eccentricity^48^. Since visual acuity (i.e., spatial resolution of the visual system) declines rapidly with the distance from the fovea^61^, these studies seem to suggest that low-resolution is sufficient for the extraction of scene gist. Consistent with this assumption, our study showed that the gist of a natural scene could be extracted in a crowding task. Given that the stimuli were presented for only 100 ms and that target scene was surrounded by four (Experiments 1 and 2) or two flankers (Experiment 3) in the current study, this finding is very striking. We postulated that the extraction of scene gist was a prioritized and largely automatic process. Therefore, we can “see the forest without representing the trees”, i.e., we can extract the gist of a scene very rapidly even before specific elements are processed^28^.

Experiment 3 investigated our hypothesis generated from Experiments 1 and 2 that the extraction of scene gist might be a prioritized process. The comparison of the extraction of scene gist to the extraction of facial expression and letter orientation in crowding tasks indeed showed an advantage for the extraction of scene gist. This finding supported our proposal that the extraction of scene gist was a prioritized process. This priority may stem from the evolutionary pressure—we are always situated in a certain scene and to survive we must respond quickly to our surroundings. This includes sensing objects (e.g., dangerous animals, faces) and motions (e.g., social actions) that provide crucial social information, even when they appear in visual periphery. Support for this proposal comes from studies showing that human can extract facial expression^24,25^, social action^62^ and scene gist (the current study) under crowding. Furthermore, rapid categorization of scene gist can even be completed by pigeons^63^. This cross-species consistency provides further support for the idea that rapid representation of scene gist is an adaptive behavior. The baseline difficulty for the three types of stimuli was not matched in this experiment. This can be an issue because task difficulty modulates crowding^64^. The gist advantage might change if the task difficulty for each stimulus type were matched. In this experiment, we tried to minimize the influence of the unmatched task difficulty by calculating a crowding strength for each type of stimuli using the performance in the uncrowded condition as the denominator.

Although the survival of high-level information under crowding has also been found with stimuli of words^22,23^ and digital numbers^26^, their stimuli were presented at only half of the eccentricity of our study and even less than half of the eccentricity of studies using faces and social actions as stimuli^3,24,62^. Thus, the present study together with others^36,48,60,63^ seems to indicate the specificity of scene gist in visual processing, probably like the specificity of faces in visual processing^54^. Brain imaging studies have shown that the contextual information in scenes consistently activates the parahippocampal place area (PPA) and retrosplenial cortex (RSC)^65–69^, suggesting a possible gist-specific pathway involving PPA and ROC for the perception of scene gist.

### Caveats

The findings of the present study confirmed our predictions, yet there are some issues that might limit the generalizability of some of these findings. First, scene gist can be crowded at both basic and global levels in presence of other scenes. Second, both low- and high-level information play roles in the crowding of scene gist, suggesting that crowding occurs at multiple levels. Third, the gist of a scene can be perceived even when it is crowded. However, it is worth noting that we did not manipulate parameters (e.g., target-flanker separation, eccentricity) to make the gist consciously inaccessible and use indicators such as priming to show that gist was still processed. Therefore, this finding may be constrained to the specific design of the present study, and caution should be taken when generalizing this conclusion to other situations. Fourth, we demonstrated that scene gist could better get through the bottleneck of crowding than facial expression and orientation of letter “E”. When drawing this conclusion, the performance in the uncrowded condition was used as the baseline, and a “crowding strength” for each task was calculated. To some degree, this measure could indicate how “crowded” a target was. However, a better approach would be equating the difficulty of the three tasks in the uncrowded condition since task difficulty modulates crowding, and then have an independent secondary measure to separate how “crowded” a target is and how much information can be extracted despite that level of crowding. In addition, only one target-flanker separation was employed in all three experiments of the present study, which may limit the inference we can make. A more appropriate approach is to systematically manipulate target-flanker separations to show participants’ performance along a continuum of target discriminability. Future research should address these issues when investigating the crowding of scene gist.

## Conclusions

The current study showed a crowding effect between adjacent scenes in the visual periphery, wherein both low-level physical properties and high-level semantic information played a role. These findings suggest that crowding of scene gist occurs at multiple stages in the visual system. It was also shown that the gist of a scene, despite being crowded, could still be greatly extracted at both basic and global levels. More importantly, scene gist is better extracted in crowding than other information including facial expression and letter orientation. Together, these findings may suggest that the extraction of scene gist is a prioritized process.

## Data Availability

The datasets generated during and/or analysed during the current study are available from the corresponding author on reasonable request.
